# Retraction Note: Discontinuation of medications classified as reuptake inhibitors affects treatment response of MDMA-assisted psychotherapy

**DOI:** 10.1007/s00213-024-06671-0

**Published:** 2024-08-10

**Authors:** Allison A. Feduccia, Lisa Jerome, Michael C. Mithoefer, Julie Holland

**Affiliations:** 1 Psychedelic.Support, Project New Day, Santa Cruz, CA USA; 2 MAPS Public Benefit Corporation, Santa Cruz, CA USA; 3 Medical University of South Carolina, Charleston, SC USA; 4 Private Practice, New York, NY USA

**Retraction Note: Psychopharmacology (2020) 238:581–588**

10.1007/s00213-020-05710-w

The Editors have retracted this article after they were informed of protocol violations amounting to unethical conduct at the MP4 study site by researchers associated with this project. The authors have subsequently confirmed that they were aware of these violations at the time of submission of this article, but did not disclose this information to the journal or remove data generated by this site from their analysis. Additionally, the authors also did not fully declare a potential competing interest. Two of the authors are affiliated with the Multidisciplinary Association for Psychedelic Studies Public Benefit Corporation (MAPS PBC), a subsidiary that is wholly owned by the Multidisciplinary Association for Psychedelic Studies (MAPS). As is stated in the Funding declaration, MAPS fully funded and provided the MDMA that was used in this trial, and MAPS PBC organised the trial.

Allison A. Feduccia agrees with this retraction but disagrees with the wording of the retraction notice. Lisa Jerome, Michael C. Mithoefer, and Julie Holland disagree with this retraction.

